# In Search for Genes Related to Atherosclerosis and Dyslipidemia Using Animal Models

**DOI:** 10.3390/ijms21062097

**Published:** 2020-03-18

**Authors:** Anastasia V. Poznyak, Andrey V. Grechko, Reinhard Wetzker, Alexander N. Orekhov

**Affiliations:** 1 Institute for Atherosclerosis Research, Skolkovo Innovative Center, 121609 Moscow, Russia; tehhy_85@mail.ru; 2 Federal Scientific Clinical Center for Resuscitation and Rehabilitation, 109240 Moscow, Russia; noo@fnkcrr.ru; 3 Department of Anesthesiology and Intensive Care Medicine, Jena University Hospital, 07743 Jena, Germany; reinhard.wetzker@uni-jena.de; 4 Laboratory of Angiopathology, Institute of General Pathology and Pathophysiology, 125315 Moscow, Russia; 5 Institute of Human Morphology, 117418 Moscow, Russia

**Keywords:** atherosclerosis, mutations, epigenetics

## Abstract

Atherosclerosis is a multifactorial chronic disease that affects large arteries and may lead to fatal consequences. According to current understanding, inflammation and lipid accumulation are the two key mechanisms of atherosclerosis development. Animal models based on genetically modified mice have been developed to investigate these aspects. One such model is low-density lipoprotein (LDL) receptor knockout (KO) mice (*ldlr^−/−^*), which are characterized by a moderate increase of plasma LDL cholesterol levels. Another widely used genetically modified mouse strain is apolipoprotein-E KO mice (*apoE^−/−^*) that lacks the primary lipoprotein required for the uptake of lipoproteins through the hepatic receptors, leading to even greater plasma cholesterol increase than in *ldlr^−/−^* mice. These and other animal models allowed for conducting genetic studies, such as genome-wide association studies, microarrays, and genotyping methods, which helped identifying more than 100 mutations that contribute to atherosclerosis development. However, translation of the results obtained in animal models for human situations was slow and challenging. At the same time, genetic studies conducted in humans were limited by low sample sizes and high heterogeneity in predictive subclinical phenotypes. In this review, we summarize the current knowledge on the use of KO mice for identification of genes implicated in atherosclerosis and provide a list of genes involved in atherosclerosis-associated inflammatory pathways and their brief characteristics. Moreover, we discuss the approaches for candidate gene search in animals and humans and discuss the progress made in the field of epigenetic studies that appear to be promising for identification of novel biomarkers and therapeutic targets.

## 1. Introduction

Atherosclerosis is a chronic vascular disease that affects large and small arteries and is characterized by the development of lipid-rich plaques in the vascular wall. The growing plaques reduce the vascular lumen and, in the case of so-called unstable plaques, can trigger thrombogenesis on the surface. The resulting ischemia can lead to fatal consequences if it affects a vital organ, such as heart [1]. During the last few decades, a significant progress has been made in the understanding of atherosclerosis pathogenesis. However, no effective treatment for the disease has been developed so far [2]. The greatest challenge is the lack of effective treatment approaches, which is the consequence of our incomplete understanding of the processes that underlie the pathogenesis. Genetic base is one of the most important features that are involved in the disease development.

Currently, the most widely used anti-atherosclerosis drugs are statins [3]. The aim of statin therapy is reducing the blood level of low-density lipoprotein (LDL) cholesterol, which is a well-known pro-atherogenic agent. It was demonstrated that statin therapy reduces the risk of cardiovascular events by approximately one third [4]. However, statin therapy alone cannot be regarded as effective treatment of atherosclerosis. Increasing the plasma levels of high-density lipoprotein (HDL), which has anti-atherogenic properties, is another promising approach. Accumulating evidence shows that high levels of HDL cholesterol can inhibit atherosclerosis progression. To date, several therapeutic agents that increase HDL levels are known, including niacin, fibrates, and statins. Among the recently developed medications are apoA-I-phospholipid complexes, human apoA-I and apoA-I-mimetic peptides, and inhibitors of cholesterol ester transfer protein, which have reached the level of clinical trials. However, the inhibitor of cholesterol ester transport torcetrapib failed to demonstrate a clinical benefit. The search for new HDL-rising therapeutic agents currently continues [5,6].

Inflammation is known to play a key role in atherosclerosis initiation and development. Ever since the concept of atherosclerosis being a chronic inflammatory condition was established, the search for anti-inflammatory agents that may be effective against atherosclerosis is ongoing. However, the complexity of signaling cascades regulating the activities of immune cells in atherosclerotic lesions makes their regulation challenging. Moreover, anti-inflammatory therapy of atherosclerosis should target lesion-specific inflammatory processes while leaving the normal immune response uncompromised [7]. Genes related to the inflammation are considered as possibly implicated in the atherogenesis, as well as genes related to the lipid metabolism.

The search for genetic determinants of atherosclerosis has been ongoing for decades. Currently, this line of research is focused on identifying the candidate genes implicated in known atherogenesis pathways and conducting association studies to evaluate their roles in the pathology development. This approach resulted in establishing the pro-atherogenic roles of numerous genes [8]. In parallel, genome-wide linkage studies were carried out to identify atherogenesis-regulating quantitative trait loci (QTL). This approach appears to be promising for identifying new atherosclerosis-related genes in an unbiased manner [9]. Finally, studies of epigenetic modifications associated with atherosclerosis present further opportunities for possible therapeutic intervention. Since the whole genome sequence information was available for humans and mice, including haplotype information, it became possible to conduct genome-wide association studies with relatively high speed.

The search for genes responsible for atherosclerosis has several challenges. First, human genome-wide association studies (GWAS) that represent a powerful modern tool for such analyses do not take into account the impact of the environmental factors and molecular processes within a particular tissue. These factors can, however, be followed using murine models. Thus, there exist currently more than 100 different strains of atherosclerotic mice with various properties’ combinations. This diversity also complicates the interpretation of findings on different models [10]. Another challenge is the difficulty of comparison of human and murine genes potentially related to atherosclerosis. Genes that were found in mouse QTL studies can be further tested in human association studies to check whether they are associated with atherosclerosis [11].

## 2. Methodology of Genetic Studies of Atherosclerosis

Every study begins with choosing the most suitable model. Despite the fact that traditionally used models of atherosclerosis greatly improved our knowledge about atherogenesis, especially in its early stages, there is currently a need for novel, more advanced models. One of the challenging areas of research is studying thrombosis associated with atherosclerotic plaque rupture, which is difficult to model in animals.

Moreover, there is a need for mouse models deficient for apoE and LDL receptors with a genetic background other than C57BL/6. However, genetic differences are present even between different substrains of C57BL/6 mice. For example, Almodovar et al. identified fourteen single nucleotide polymorphisms (SNPs) resulting in differences between the two most popular lines from Jackson Laboratory and Taconic [12]. Moreover, the Nnt deletion on chromosome 13 was shown to be different between these two lines. At the phenotypic level, these differences resulted in variability of adiposity between the substrains that were relevant for atherosclerosis research. Therefore, differences in the genetic background within one line may cause reproducibility problems and also affect the testing parameters in an unpredictable way.

It is clear by now that the atherosclerosis is a polygenic disorder, and it is necessary to use models with various genetic backgrounds to identify the genetic features that are responsible for the disease development with confidence confidently. Several works have been conducted to assess the genetic variability between mouse strains. Grainger et al. performed an investigation on intercrosses of BALB/cJ (BALB) and SM/J (SM) apolipoprotein E-deficient (*apoE^−/−^*) mice to identify chromosomal regions harboring genes contributing to carotid atherosclerosis. With the use of QTL analysis and bioinformatics tools, they found out that there are five significant QTL, among which the one on the chromosome 12 had the highest LOD score. Potential candidate genes were listed: *Arhgap5, Akap6*, *Mipol1*, *Clec14a*, *Fancm*, *Nin*, *Dact1*, *Rtn1*, and *Slc38a6* [13]. Another study, performed by Wang et al., engaged NOD (non-obese diabetic) mice. They found that the knockdown of *ldlr* or *apoE* in NOD mice did not lead to atherosclerosis development. By contrast, C57BL/6 mice with the ApoE deficiency developed the disease when fed with a high-fat diet. Moreover, simultaneous knockdown of both *ldlr* and *apoE* resulted in a severe atherosclerosis [14].

However, the large majority of the mouse models of atherosclerosis are currently created on the C57BL/6 genetic background. This limits their mapping power and coverage of allelic diversity. QTL analysis on F2 intercrosses of SM/J- *apoE^−/−^* and BALB/cJ- *apoE^−/−^* mice revealed that *tnfaip3* was the most potent causal gene. These results show the importance of the use of different strains in the studies aimed at identification of the genetic causes of the atherosclerosis [15].

Creation of mouse models of atherosclerosis allows for revealing new atherosclerosis-related QTL [11]. Such identification may, in its turn, identify previously unknown atherogenesis pathways and new potential therapeutic targets. Although human and mouse orthologs of atherosclerosis-related genes do not always coincide, a search can be conducted for other players of the identified pathway that may prove to be useful in that regard.

Inflammation and lipid metabolism deregulation represent good entry points for searching for relevant atherosclerosis-related genes. However, due to the crucial role of many of these genes for reproduction and survival, they have been subject to great selective pressure and therefore their pro-inflammatory polymorphisms can be found easily. To date, six genes involved into the inflammatory response were identified: *ALOX5AP* [16] and *MEF2A* [17] in human linkage studies of myocardial infarction, *Alox5* [18] and *Tnfsf4* [19] in mouse linkage studies of atherosclerosis and *LTA* [20] and *PSMA6* [21] in human genome-wide association studies of myocardial infarction (Table 1) [17,18,19,20,22,23]. The obtained results indicate that genetic predisposition to inflammation may account for a considerable part of variance of atherosclerosis incidence in populations [9].

Despite the progress made, identification of atherosclerosis-related genes by means of linkage studies remains difficult. One of the challenges is the complexity of atherosclerosis pathogenesis: the involvement of different cell types in the pathological process, the heterogeneity of genes implicated in the process, and the fact that each of them by themselves may have only a small effect, and the role of the environmental factors. As a result, most genetic variants that were identified in genome-wide association studies have not been previously captured in QTL. Moreover, genes responsible for a certain trait tend to cluster within the chromosome and interact with each other, making positional cloning difficult.

It has been demonstrated that not all genes that influence the phenotype of the QTL may be detected by positional cloning [18]. Other QTL genes can be found by breaking down congenic regions to smaller chunks and analyzing them separately. Multiple causal QTL genes can be identified by association studies conducted for genes in the QTL peak, also called “peak wide mapping” [24]. The chance of identifying human atherosclerosis genes can potentially be increased by using the mouse–human comparative genomic approach. Whether a particular gene is associated with the increased risk of atherosclerosis can be determined by candidate gene association studies that are, however, limited by the high frequency of false-positive results. Moreover, this approach for gene searching is biased, since only genes that are suspected of being related to atherosclerosis are tested. Even the established associations provide little information on whether and how the polymorphisms are linked to the altered gene functions. Genome-wide association studies were quite successful in detecting unexpected candidate atherosclerosis-related genes, but could not be used widely enough due to high costs and complexity of the procedures.

Microarray and genotypic methods offer the possibility of quantifying the expression of numerous (up to thousands) of genes simultaneously in an unbiased way. These methods allow for locating QTLs for phenotypes based on the expression data (so called expression QTLs, or eQTLs). To identify candidate genes for further studies, a combination of clinical QTL with eQTL can be used [25].

Bone marrow transplantation can be considered as an additional approach to identifying the pro-atherosclerotic roles of specific genes in mice. For instance, this method allowed for demonstrating the impact of hematopoietic-expressed genes on atherosclerosis [26].

Each of the approaches listed above has its limitations and advantages. The most promising strategy for finding new genes that are responsible for atherosclerotic development appears to be combining more than one approach in a single study.

## 3. Genetic Aspects of Atherosclerotic Disease in Mice

Mouse is the most commonly used model animal for studying human diseases, including atherosclerosis. First attempts to create a suitable mouse model of the disease were based on dietary modifications. Wild type mice that are fed with a regular chow diet do not develop atherosclerosis spontaneously. For that purpose, special diets have been developed, such as high fat (15%), cholesterol (1.25%), and cholate (0.5% cholic acid) diet, or “Paigen Diet”. This diet is commonly used to induce atherosclerosis lesions in wild type animals. The diet is characterized by a reduced conversion of cholesterol into bile acid, and impaired cholesterol clearance, which leads to increased plasma cholesterol. Moreover, cholate-rich diet alters the expression of several genes that are responsible for lipid metabolism and inflammatory response [27].

More recently, the development of new genetic tools allowed for creating genetically modified mouse models of human atherosclerosis that allowed for better accuracy in reproducing human atherosclerotic lesions. First, mice deficient for low density lipoprotein receptor (*ldlr*^−/−^) were created. These animals lack the primary receptor responsible for LDL cholesterol (LDL-C) uptake, which results in its increased plasma concentration. Further increase of LDL-C in these animals is achieved by feeding them with a Western-type diet (WTD). Another approach is to cross the *ldlr*^−/−^ mice with another mutant strain, such as *apobec1* knock-out mice. Mice deficient for apolipoprotein-E (*apoE*^−/−^) represent another atherosclerosis model, which is currently widely used. In these animals, circulating lipoprotein contains no primary ligand that is used for facilitating the lipoprotein uptake through hepatic receptors. This results in a plasma cholesterol increase that is more prominent than in *ldlr*^−/−^ mice, with spontaneous development of atherosclerotic lesions in animals fed with a regular diet and further increase of the disease induced by the WTD [28].

Mouse models of atherosclerosis proved to be very helpful for genetic studies of the disease. Over 100 mutations involved in the pathogenesis of atherosclerosis have been identified in mice. Many of these genes may be involved in common pathways in atherosclerosis development [29]. However, only a few of these identified genes were demonstrated to play a causative role in human coronary artery disease. A possible explanation for this is the high selection pressure on these genes that prevents loss-of-function mutations to spread in the human population. Another reason is the differences in the pathogenesis mechanisms of atherosclerosis in humans and mice. Such differences may include many aspects, starting from the size, structure, and elasticity of the arteries and ending with distinct mechanisms of complicated plaque formation, which are difficult to reproduce in mice. Moreover, atherosclerotic disease affects different arteries in humans and mice, with coronary, carotid, and cerebral arteries being the most important in humans, with aorta and proximal large vessels being the first affected in mice [30].

The translation of the genetic findings from mouse models to human satiation was slow and unconvincing. This resulted not only from the limitations of mouse models, but also from the insufficient development of human genetics to identify significant genetic associations with atherosclerotic disease.

## 4. Candidate Gene Approaches in Mice

**Figure 1:**
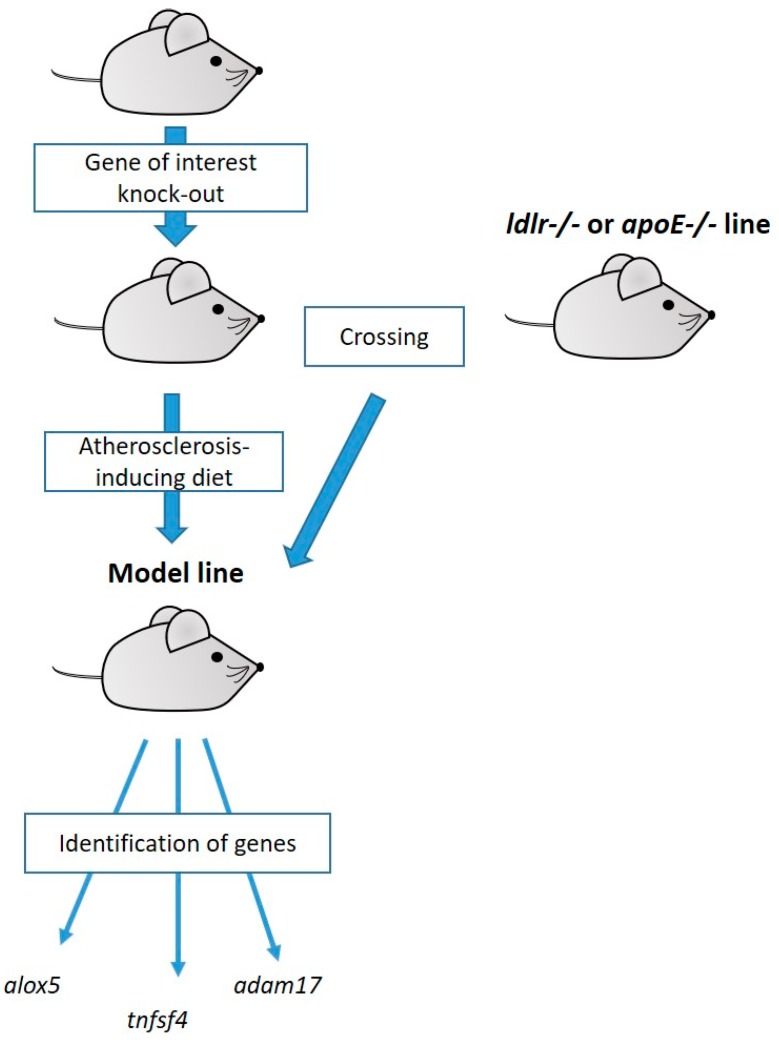
The scheme of a candidate loss-of-function approach and the atherosclerosis-related genes it helped to identify.

Two main approaches have been used for candidate atherosclerosis-related gene testing in mice. The first method, a candidate gene loss-of-function approach, involves the creation of genetically-modified animals deficient for a specific gene with subsequent induction of atherosclerosis either by crossing the mouse line of interest with *ldlr*^−/−^ or *apoE*^−/−^ mice or by using the atherosclerosis-inducing diet (Figure 1). This approach allowed identifying associations of more than 100 genes with atherosclerotic disease in mice. About 60% of these genes were initially studied in mice with *apoE*^−/−^ genetic background, around 25% in mice with *ldlr*^−/−^ background, and 10% in animals with diet-induced disease. Many of the tested candidate genes had no or only minimal effect on atherosclerosis development. Some genes only had an effect in a certain genetic background; for instance, *icam1*^−/−^ had an effect only in *apoE*^−/−^ (but not *ldlr*^−/−^) mice. A QTL analysis allowed mapping 43 significant loci, of which 19 were identified in *apoE*^−/−^ animals, 12 in *ldlr*^−/−^ animals, and 12 in the diet-induced model [15].

The gene of arachidonate 5-lipoxygenase (*alox5*) was shown to be linked to atherosclerosis in mice, since animals deficient for this gene developed atherosclerotic phenotypes. However, genetic studies in humans have not confirmed the association of *ALOX5* gene with coronary artery atherosclerosis. At the same time, another human atherosclerosis-associated gene, *CXCL12*, could be mapped to the orthologous mouse chromosome Chr6, at a distance of less than 1 Mb from *alox5,* indicative of a potential contribution of *cxcl12* polymorphisms to the Artles QTL [31].

Another gene potentially causally related to atherosclerosis is *tnfsf4*, which was identified in Ath1, one of the first atherosclerosis-associated QTLs mapped in mice. This gene, also known as Ox401, is a member of the tumor necrosis ligand superfamily. Mice deficient for this gene demonstrated decreased atherosclerosis progression. Moreover, studies in humans have revealed SNP that were related to myocardial infarction with a marginal significance [32], which was, however, not confirmed by other studies in humans [33]. The human ortholog *TNFSF4* has no known association locus in its proximity. A disintegrin and metalloproteinase 17 (*adam17*) has been proposed as another gene causally related to atherosclerosis, but the effect of its knock-out remains to be investigated [34].

## 5. Genetics of Atherosclerosis in Humans

Atherosclerosis is a multifactorial disease involving a range of biological mechanisms, from lipid metabolism disruption to inflammation. It is therefore unlikely to pinpoint a unique gene that is responsible for disease initiation. However, a range of causal genes acting together can probably be identified and used for finding potential therapeutic targets. In humans, numerous genes have been shown to be important for the individual’s susceptibility to atherosclerosis. Genetic variants and mutations that increase the risk of atherosclerosis and related cardiovascular diseases were identified. If each of these variants would have relatively little effect in the overall balance, their combination with each other and with the genetic background may have pronounced effects. Genetic studies in humans are challenging because of the genotype complexity, limited sample sizes, and heterogeneity of the observed phenotypes.

## 6. Candidate Gene Studies in Humans

Candidate gene studies allow for revealing associations of individual gene polymorphisms with atherosclerosis. To date, several genetic loci that are likely to play a role in the disease pathogenesis have been revealed [35]. However, a large part of candidate gene studies has not yielded convincing results or reported associations were weak and impossible to confirm. Some progress was achieved after development of a cardiovascular gene-centric 50K SNP array, which allowed for revealing several new genes with a significant association with coronary artery disease [36].

In general, genetic markers are less powerful predictors of atherosclerosis than traditional risk factors, such as gender, age, or behavioral factors. However, more detailed studies, including studies on twins, have revealed the existence of genetic susceptibility to atherosclerosis. In rare cases, such as familial hypercholesterolemia (FH), atherosclerosis can be inherited following Mendelian laws. In FH, mutations in the *LDLR* gene lead to a prominent increase of plasma LDL cholesterol level, which strongly increases susceptibility to atherosclerosis [37]. The genetic variants leading to FH-associated atherosclerosis have been studied using a QTL approach in several large families and by studying a single large family under a Mendelian inheritance hypothesis. QTL mapping in humans encounters the same challenge as it does in mice: the large size of the linkage regions that hinders the causal genes identification [9]. This may explain the small number of the identified atherosclerosis-associated genetic loci that could be confirmed by several studies.

The identified atherosclerosis-associated candidate genes include lipoprotein receptor-related protein 6 (*LRP6*) at 12q13.2, arachidonate 5-lipoxygenase-activating protein (*ALOX5AP*) at 13q12-13, and myocyte enhancer factor 2A (*MEF2A*) at 15q26.3 [38]. These genes have not been identified in studies on mouse models. Further studies using a genome-wide association approach have identified new loci outside of the original confidence intervals for *LRP6* and *ALOX5AP*, indicating that these may be distinct loci. At the same time, *MEF2A* may be linked to a broader region on chromosome 15 that includes another gene, *ADAMTS7* [29].

Genome-wide association studies, or GWAS, represent a powerful tool that makes possible simultaneous investigation of millions of polymorphisms to reveal their association with a certain phenotype within a large population [39]. Studies using GWAS have revealed several human genetic loci strongly associated with coronary artery disease and myocardial infarction [40]. Other examples of large GWAS are the CARDIoGRAM consortium that has analyzed data from 14 different GWAS for a total of 140,000 patients, and reported 13 new coronary artery disease-associated loci and the C4D consortium that investigated 70,000 patients from South Asia and Europe and identified four new loci [41,42]. Apart from the loci mentioned above, numerous other loci were identified that may be significant (*p* < 0.05, but higher than the significance threshold currently used for GWAS, which is 5 × 10^−8^). It is possible that further research will demonstrate significance of some of these loci in a larger meta-analysis.

GWAS helped to reveal the association between several genes implicated in triglyceride metabolism and cardiovascular disease, including *APOA5* and *APOC3* [43]. Rare *APOA5* mutations were shown to be associated with enhanced plasma triglyceride levels and, at the same time, the elevated risk of coronary artery disease [44]. By contrast, rare loss-of-function mutations in the *APOC3* gene were demonstrated to lower both the plasma triglyceride levels and the cardiovascular risk [45]. These findings make the *APOC3* and *APOA5* the promising lipid metabolism-related target for future development of atherosclerosis and dyslipidemia treatment approaches.

Loss-of-function variants of another gene related to the triglyceride metabolism, *ANGPTL3*, were revealed to be atheroprotective due to their association with the decrease in plasma levels of triglycerides, LDL cholesterol, and HDL cholesterol [46]. Rare variants of *HSD17B13* were found to be significantly associated with triglycerides and HDL in white individuals with type 2 diabetes. This provided an explanation for the observed lipid variation in response to fenofibrate treatment in individuals with type 2 diabetes treated with statins [47].

## 7. Epigenetic Factors

Epigenetic factors play important roles in many human diseases, including atherosclerosis. They are increasingly recognized as disease modifiers and potential therapeutic targets. Among the known epigenetic factors are DNA methylation, histone modification, and the effects of various non-coding RNAs. Studying of epigenetic factors is challenging because of their complexity and dynamic nature, but also by the limited access to in vivo material and tissue heterogeneity, since epigenetic modifications are often cell type-specific [48]. These limitations make single cell analysis technologies. Single cell RNA sequencing allowed revealing disease stage-specific markers with subsequent isolation of specific cell population suitable for epigenetic profiling. Combined with modern computational strategies for data analysis, these approaches can help with revealing gene regulatory networks for a particular cell type that are present in atherosclerotic plaques [49].

In contrast to genetic, epigenetic modifications have a dynamic nature. They can be influenced by the environmental stimuli that can modify the cell phenotype, gene expression patterns, and the expression and regulation of the transcription factors [50]. For instance, it was demonstrated in mice that transplantation of macrophages into a new tissue environment changed the epigenetic profiles and gene expression patters and rendered the transplanted cells phenotypically similar to tissue-resident macrophages [51].

### 7.1. DNA Methylation

DNA methylation is performed by methyltransferases (DNMT) that add methyl groups to the 5′ position of cytosine rings in the CpG dinucleotides. Insufficient DNA methylation leads to gene activation, and hypermethylation to gene silencing [52]. Recent studies revealed significant levels of DNA hydroxymethylation within genes characterized by active transcription, as well as in their enhancer regions [53].

Recent studies have demonstrated that, in both humans and mice, overall DNA hypermethylation of CpG islands is observed in atherosclerosis [54]. Moreover, genome-wide DNA methylation sequencing revealed a positive correlation between DNA methylation status and atherosclerotic lesion grade [55]. It is therefore likely that DNA methylation plays a role in atherosclerosis development, and therefore a DNA methylation status can potentially be used as an atherosclerosis biomarker.

### 7.2. Histone Modification

Histones are highly alkaline proteins that play a major role in nucleosome formation. Histone modifications are represented by a complex of covalent post-translational modifications, such as phosphorylation, methylation, and acetylation. Histones can also be ubiquitinated and SUMOylated [56]. Chromatin-remodeling complexes are capable to control histone modifications in a dynamic way [57]. The process is carried out by ‘writer’ and ‘eraser’ complexes that introduce or remove covalent modifications of lysine or, less frequently, arginine residues of histone proteins.

### 7.3. Long Non-Coding RNAs

Long non-coding RNAs (lncRNAs) are commonly defined as RNA transcripts shorter than 200 nucleotides that do not encode a functional protein. LncRNAs are polyadenylated, contain only 2–3 exons, and are typically spliced [58]. Most of them are transcribed by polymerase II. LncRNAs are associated with the regulations of numerous cellular processes in mammals, such as chromatin remodeling, chromatin modification, dosage compensation effect, genomic imprinting, and others [59]. These molecules do not act by themselves, but form complexes and execute their regulatory functions through interacting with proteins. LncRNAs were shown to interact with different enzymes and complexes and thus affect DNA methylation, histone methylation, and acetylation. They also are able to regulate the transcription by the interaction with transcriptional factors. The implication of lncRNAs in post-transcriptional regulation is based on their ability to interact with splicing factors and proteins and subsequently regulate mRNA alternative splicing, and splicing factors can also directly regulate lncRNA alternative splicing [59].

However, lncRNA are better to define not as non-coding, but as likely to be non-coding, because it is impossible to verify the absence of coding properties with certainty. The most significant piece of criteria used to distinguish whether the transcript encodes a peptide is the lack of open reading frames (ORFs). The first attempt to identify lncRNAs and to summarize the data available from studies in mice was the FANTOM project [60]. It used the cDNA cloning with the subsequent Sanger sequencing.

Although comprehensive data on lncRNAs remain to be collected, there is growing evidence that lncRNAs play a role in epigenetic and/or transcriptional processes through recruiting chromatin modifying and transcriptional factors to DNA among other mechanisms [61]. It was also shown that lncRNAs can participate in post-transcriptional regulators by controlling translation, splicing, and mRNA stability [62].

In cardiovascular diseases, several lncRNAs have been identified as epigenetic regulators playing a role in the pathological processes. For instance, the antisense non-coding RNA in the Ink4 locus (ANRIL) that is transcribed from the human 9p21.3 locus, the expression of which is strongly associated with the incidence of coronary artery disease [63,64]. It was shown that ANRIL could promote proliferation of human vascular smooth muscle cells through recruiting repressive PcG protein complexes to the cell cycle inhibitor genes *CDKN2A/B* [65]. In the endothelial cells, ANRIL was shown to be induced by NF-κB signaling. In this cell type, it can induce the expression of pro-inflammatory genes IL-6 and IL-8 through recruiting the transcription factor YY1 [66].

Another lncRNA that was shown to be involved in atherosclerosis development is lincRNA-p21. In atherosclerotic plaques from *apoE^−/−^* atherosclerosis mouse model and from human coronary arteries, this lncRNA was found to be downregulated. Moreover, knock-down of lincRNA-p21 was demonstrated to increase the neointima growth in a mouse model of carotid artery vascular injury. This process was partially dependent on p53-dependent apoptotic genes regulation in vascular smooth muscle cells [67]. Finally, several lncRNAs were also found to be involved in such processes as inflammation and innate immunity regulation, which makes them relevant for atherosclerosis research [68,69]. Future studies are likely to add to the growing list of lncRNAs implicated in atherosclerosis and evaluate their potential as biomarkers or even therapeutic targets.

## 8. Conclusions

The complexity of atherosclerosis pathogenesis makes it impossible to identify a precise set of genes responsible for the disease development. Instead, multiple genes have been identified that can participate in different stages of atherosclerosis progress. Finding genetic determinants for atherosclerosis can crucially improve not only general understanding of underlying processes, but also an individualized medication. The same symptom was formed by environmental factors, genetic predisposition or their combinations in different patients, and these patients may call for different therapies.

There are three main challenges impeding studies in the field of finding genetic and epigenetic determinants for atherosclerosis. The first one is to establish a suitable model because of the complexities in translating results obtained from classical models to humans and because of serious limitations of using human material. The second challenge is to create an economically viable and unbiased approach that can be widely used in order to make data be able to be reproduced and standardized. Despite the general understanding of how to target the particular gene in the case of therapeutic strategy, the further elaboration of a genetically-based treatment or preventive approach for atherosclerosis also remains challenging.

## Figures and Tables

**Table 1:** Brief summary on six important genes potentially involved in atherosclerosis development.

Name	Gene ID	Link to Atherosclerosis	Normal Function	Identification (Organism, Method)	Reference
*ALOX5AP*	241	Genetic variants are potentially contributed to the higher coronary heart disease risk	Part of leukotriene biosynthesis pathway	Human, haplotype association study	[22]
*MEF2A*	4205	MEF2A signaling pathway is involved in pathogenesis of familial CAD and MI	Myocyte-specific transcription factor	Human, linkage study	[17]
*Alox5*	11689	homozygote 5LO null mice develop smaller atherosclerotic lesions	Part of leukotriene biosynthesis pathway	Mouse, linkage study	[18]
*Tnfsf4*	22164	blocking the OX-40/OX40L interaction reduced atherogenesis	OX40 ligand	Mouse, linkage study	[19]
*LTA*	4049	Genetic variants are potentially contributed to the higher MI risk	Lymphocyte cytokine	Human, GWAS	[20]
*PSMA6*	5687	PSMA6 rs_1048990 polymorphism may contribute to MI susceptibility in type 2 diabetes	-	Human, GWAS	[23]

CAD: coronary artery disease; MI: myocardial infarction; GWAS: genome-wide association study.
